# Resting connectivity predicts task activation in pre-surgical populations

**DOI:** 10.1016/j.nicl.2016.12.028

**Published:** 2016-12-24

**Authors:** O. Parker Jones, N.L. Voets, J.E. Adcock, R. Stacey, S. Jbabdi

**Affiliations:** a FMRIB Centre, NDCN, University of Oxford, John Radcliffe Hospital, Headington, Oxford OX3 9DU, UK; b Oxford Epilepsy Research Group, NDCN, University of Oxford, John Radcliffe Hospital, Oxford OX3 9DU, UK; c Department of Neurology, Oxford University Hospitals NHS Trust, John Radcliffe Hospital, Headington, Oxford OX3 9DU, UK; d Department of Neurosurgery, Oxford University Hospitals NHS Trust, John Radcliffe Hospital, Headington, Oxford OX3 9DU, UK

**Keywords:** Connectivity, Individual variation, Neural pathology, Resting-state fMRI

## Abstract

Injury and disease affect neural processing and increase individual variations in patients when compared with healthy controls. Understanding this increased variability is critical for identifying the anatomical location of eloquent brain areas for pre-surgical planning. Here we show that precise and reliable language maps can be inferred in patient populations from resting scans of idle brain activity. We trained a predictive model on pairs of resting-state and task-evoked data and tested it to predict activation of unseen patients and healthy controls based on their resting-state data alone. A well-validated language task (category fluency) was used in acquiring the task-evoked fMRI data. Although patients showed greater variation in their actual language maps, our models successfully learned variations in both patient and control responses from the individual resting-connectivity features. Importantly, we further demonstrate that a model trained exclusively on the more-homogenous control group can be used to predict task activations in patients. These results are the first to show that resting connectivity robustly predicts individual differences in neural response in cases of pathological variability.

## Highlights

• A method for identifying eloquent areas in the brain from resting fMRI is proposed.
• It uses supervised learning to predict task contrasts from resting connectivity.
• Good predictions were obtained in controls and in pre-surgical patient populations.
• Patient diagnoses included epilepsy, tumours, and vascular lesions.
• Language maps in patients could be predicted from models trained on controls.

## 1 Introduction

When presented with the same task, each person's brain tends to respond in an idiosyncratic way (Penfield and Roberts, 1959, Ojemann et al., 1989). From the point of view of task-based fMRI, these *individual differences* can be seen as distinct spatial patterns of neural activation. Although there are strong similarities between individual brains, which allow for group studies to be carried out, group averages lack the specificity of single-subject fMRI. This is critical for the use of fMRI in pre-surgical planning where the degree of individual variability is typically higher in patients compared to non-surgical controls. Single-subject task-based fMRI therefore provides a valuable tool for the identification of neural tissues associated with key functions such as language processing (Binder et al., 1997, Price, 2012), particularly as the exact location and extent of higher-order cortical areas cannot, in general, be determined from gross anatomy (Fischl et al., 2008). One specific challenge is that task-based fMRI requires subjects to perform experimental tasks. However, not all pre-surgical patients are able to perform these tasks—for reasons that range from patient compliance or fatigue, to the ability to perform or even comprehend the task instructions. Furthermore, the choice of task can be limited by constraints on scan time. For patients who cannot perform the relevant task, a ‘task-free’ method for mapping brain functions would therefore fill an important gap. In this paper, we ask whether individual variation in activation maps can be predicted for a clinically-relevant language task in pre-surgical patients, using only resting-state fMRI and with no explicit experimental task.

**Fig. 1:**
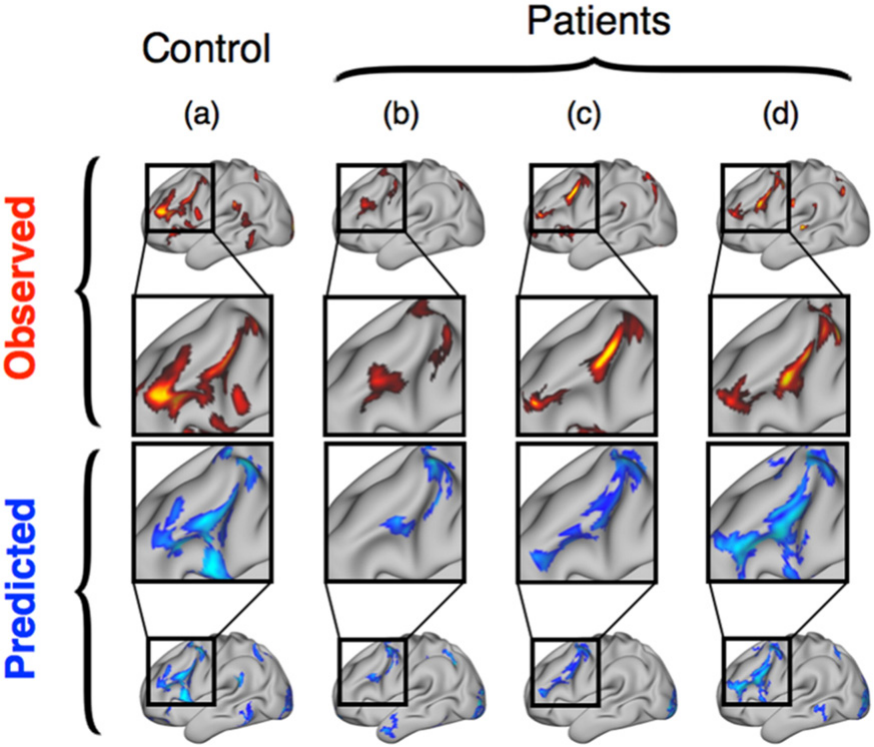
Individual task-contrasts can be predicted from connectivity measures derived from resting-state fMRI. Above: observed task-contrasts (red) for the category-fluency task in four subjects (three patients). Below: predicted task-contrasts (blue) generated using each subject's resting scan (results from the leave-one-out analysis). Predictions best resemble their paired observations both in healthy and in pathological cases.

We acquired resting-state and task-evoked fMRI from 71 pre-surgical patients and 32 healthy controls (103 subjects total). The pre-surgical patients had been diagnosed with conditions that represent potential use cases: operable brain tumours, temporal lobe epilepsy, and vascular lesions (specifically, arteriovenus malformations and cavernomas). We acquired task-based fMRI to infer individual task activation maps which were then used to both develop/train and evaluate our model predictions. For the task we used *category fluency*, which is known to activate language-processing areas in the inferior frontal cortex using the contrast [category fluency > fixation baseline] (Paulesu et al., 1997, Costafreda et al., 2006). Healthy volunteer subjects were included in the study to compare the variability of task activity with that of patients, and to compare within-group and between-group predictions. The predictions were produced using a supervised approach (see Methods). Briefly, the resting-state data were pre-processed into individual subjects' resting-connectivity features, which were then used to train a set of regression models to predict whole-brain task activation maps. This allowed us to produce, from their resting-connectivity features alone, the unseen test subjects' *predicted* task activation maps (Fig. 1).

In prior work, we applied this method to predict task activation maps from resting-connectivity features using healthy control data from the Human Connectome Project (Tavor et al., 2016). In that paper, we were able to predict high-fidelity task-activation maps across a wide range of task domains. Important questions however remained unanswered, the most critical being whether our approach could be usefully applied in a clinical setting, given shorter, lower quality data sets and given greater individual variability in patients than in controls. These questions are tackled in the present paper; in addition, we explore the possibility of training a single model on data from healthy controls alone, and then using the model to predict each patient's task activity. This ‘transfer-learning’ would make it possible, in theory, to predict activity for additional task domains without the need to acquire task-based fMRI training data in patients, potentially opening the doors to a novel and powerful approach to pre-surgical mapping.

## 2 Materials and methods

### 2.1 Subjects

All subjects gave informed consent prior to participating in the study, which was approved by the London Surrey Borders Research Ethics committee.

We report data from 103 subjects: 71 patients and 32 healthy controls. The patients underwent language mapping as part of the study and no patients recruited were excluded from our analyses. Patients presented with pathology in frontal and temporal lobes and were being considered for neurosurgery. The pathologies were: temporal-lobe epilepsy (TLE), brain tumour (TUM), cavernoma (CAV), arteriovenous malformation (AVM), and focal cortical dysplasia (FCD). Because there were relatively few cases of CAV, AVM, and FCD, we combined these into one ‘other’ group (n = 9). The healthy controls (CON) were selected to match the distribution of sex, age, and handedness in the patient group. All subjects had normal or corrected-to-normal vision. This was important as the experiment required subjects to read task instructions from a visual prompt.

In addition to information on sex, age, and handedness, a subset of patients (n = 33) further participated in an intracarotid sodium amobarbital procedure (‘Wada test’). Another subset of 51 patients provided out-of-scanner behaviour scores for the category fluency task (see Table 1).

### 2.2 In-scanner behaviour

Subjects performed a (covert) category fluency task in scanner. This involved subjects receiving semantic categories as cues before imagining as many nouns in that category as possible. The categories presented were animals, tools, countries, vehicles, and fruits. Categories were presented as written words on a screen viewable in-scanner. Before beginning the experiment, subjects were instructed to respond covertly, that is imagining their responses but without speaking aloud. Outside of the scanner, all subjects also completed an overt fluency task for the category vegetables, from which behavioural scores were recorded for 51 patients. We used a block design with 5 repeats of 30 s in the active condition (task) and 30 s in an inactive condition (baseline).

### 2.3 Image acquisition

All imaging data were acquired at Oxford's Centre for Functional Magnetic Resonance Imaging of the Brain (FMRIB) on a 3T Siemens Verio MRI scanner equipped with a 32-channel head coil. We acquired anatomical *T*_1_-weighted structural scans using MPRAGE and functional *T*_2_*-weighted scans using an EPI sequence. One high-resolution structural scan was acquired per subject at a resolution of 1 mm isotropic voxels (174 × 192 × 192 total voxels). In the resting fMRI (rfMRI) sessions, we collected 85 volumes (TR = 3.5 s; TE = 30 ms) from each subject at a resolution of 2 mm isotropic voxels (54 slices, 96 × 96 matrix), and in the task fMRI (tfMRI) sessions we collected 101 volumes (TR = 3 s; TE = 28 ms) at a final resolution of 3 mm isotropic voxels (44 slices, 64 × 64 matrix). Each subject spent 5 min on an rfMRI session and 5 min on a tfMRI session. We also created lesion masks for the 27 relevant patients with large AVMs, CAVs, or TUMs, which were defined on the T1w anatomical scan manually (N.L.V.).

### 2.4 Preprocessing

Functional data were brain-extracted using BET (Smith, 2002), temporally high-pass filtered at 100 s, motion corrected using MCFLIRT (Jenkinson et al., 2002), spatially smoothed at 4 mm FWHM (full-width half maximum), linearly co-registered to individual structural scans with BBR (Greve and Fischl, 2009), and non-linearly registered to MNI152 space with a 10 mm warp resolution with FNIRT (www.fmrib.ox.ac.uk/fsl/fnirt). A contrast of interest was defined for task > baseline and modelled in FEAT using a boxcar design with double-gamma HRF (hemodynamic response function), temporal derivatives, and temporal filtering. The resulting volumetric data were then projected onto a standard surface using trilinear interpolation in Connectome Workbench (http://www.humanconnectome.org), producing data on 91,282 ‘grayordinates’ that included cortical surface vertices and subcortical voxels (Glasser et al., 2013).

### 2.5 Feature extraction

The features that we input into the model were individual-level resting-state network maps, which were derived from individual rfMRI data through Dual Regression as described by (Filippini et al., 2009).

Before the Dual Regression step, we first defined a set of group-level connectivity features in the following way. Independent data (rfMRI scans) were selected for 100 random subjects from the minimally pre-processed HCP database (Q3 release). These data were high-quality rfMRI scans acquired over four 15-min sessions (for a total of 1 h) per subject (Glasser et al., 2013).

Next we applied incremental PCA to the time series datasets in order to reduce the data dimensionality (Smith et al., 2014) (keeping 1000 dimensions), and then used group-ICA to factorise the concatenated data into a set of 40 spatial components per hemisphere. We excluded components that did not replicate in both hemispheres, as a way to reduce the number of visible artefacts. This resulted in a final set of 33 group-level connectivity features (Beckmann et al., 2005).

We then used these group-level results in a Dual Regression analysis (Filippini et al., 2009) in order to infer individual-level versions of the group ICA maps for all 103 subjects (patients and controls).

In the first Dual Regression step, each subject's rfMRI time-series data was regressed against the group-level ICA maps (multiple regression), which produced a set of individual time courses × components. We then regressed each subject's rfMRI time-series (single regressions) against their individual time courses × components, in the second regression step, in order to produce a set of components × individual spatial maps. Unlike Tavor et al. (2016), here we used single regressions in the second step of the dual regression, due to the lower number of time points. The resulting 103 spatial maps were normalised to zero mean and unit norm and then used as individual connectivity features, which were input to the predictive model.

### 2.6 The predictive model

A simple piecewise linear-regression approach was used to predict spatial task-activation maps. In the first training regime, we used leave-one-out cross-validation to test on each subject. In the second regime, we split the data into controls and patients, training on the controls and testing on the patients (transfer analysis).

For each subject in the training set, *i*, we paired *i*'s input features **X**^(*i*)^ (an *n* × (1 + 33) design matrix where the first column models the intercept and n = 91.282) and *i*'s observed n × 1 task activation map **y**^(*i*)^. The regression coefficients **β**^(*i*)^ were then inferred analytically:

$$\beta^{\left(i\right)}=\text{pinv}\left(X^{\left(i\right)}\right)∙y^{\left(i\right)}$$

For any test subject *j*, we generated a predicted task activation map ${\hat{y}}^{\left(j\right)}$ from *j*'s input features **X**^(*j*)^ and from an estimated model coefficient $\hat{\beta }$:

$$\;{\hat{y}}^{\left(j\right)}=X^{\left(j\right)}∙\hat{\beta }$$

In the leave-one-out regime, the estimated model coefficients $\hat{\beta }$ were derived from the mean of all *m* − 1 individual **β** values, where test subject *j*'s actual **β** value was excluded:

$$\hat{\beta }=\frac{1}{m-1}\sum_{i=1:i\neq j}^{m}\beta^{\left(i\right)}$$

For the analysis that trained on controls and tested on patients, $\hat{\beta }$ was just the mean of all 32 control subjects' **β**s.

Although it is possible to invert the full rank 91,282 × 34 matrix, we found this worked less well than using a piece-wise linear approach, in which the brain is first parcelled into non-overlapping parcels and then linear models are fitted and predictions generated within each parcel separately. The resulting predictions can be concatenated to produce a predicted task activation map for the whole brain.

The parcels we used were derived using group-ICA on 100 rfMRI scans taken from an independent dataset: the HCP (see section on Feature extraction). We used winner-takes-all on a set of 50 ICA components, so that each vertex was assigned to exactly one of the 50 components. These 50 cortical parcels ranged in size from 316 to 6771 vertices (see Supplementary Fig. 1).

### 2.7 Model evaluation

For each subject, we asked how well the prediction matched the observed-task activation map by calculating the Pearson correlation coefficient between actual and predicted spatial maps.

**Fig. 2:**
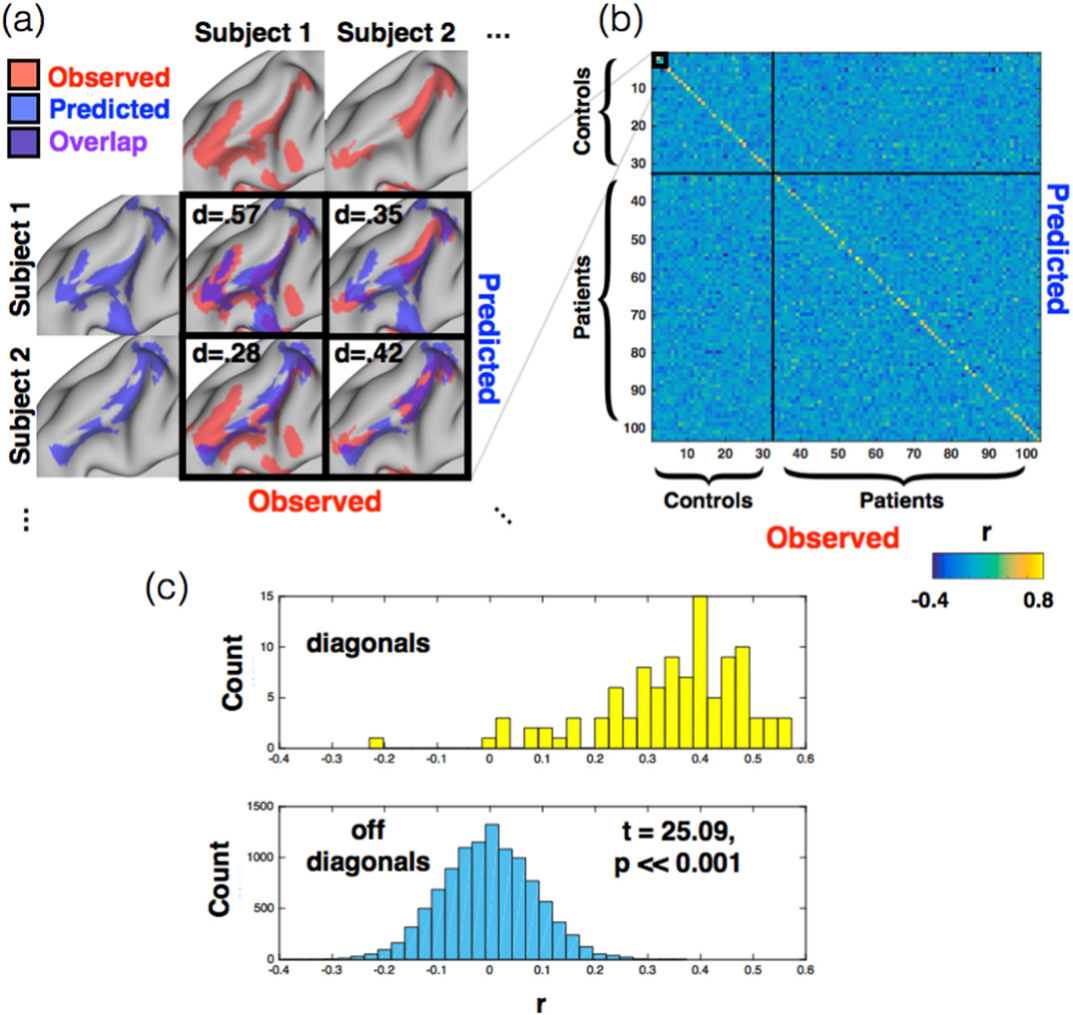
Model predictions correlate with observed activations across subjects. (a) Visualising the overlapping activation between two example subjects' maps (i.e., subjects (a) and (c) from Fig. 1). For illustrative purposes, these maps have been thresholded and binarised. Binary overlaps were quantified using the Dice coefficient, which is highest along the diagonal (representing each subject's observed and predicted maps). The off-diagonals pair different subjects' maps. (b) The pattern in (a) generalises across most subjects, producing a similarity matrix of correlated maps. The strong diagonal shows that similarity is strongest within subjects' maps. Pixel intensities show correlations (Pearson coefficients); these have been normalised across the rows and columns to make the results comparable between scans. (c) Histograms of the correlations in (b). The off-diagonals (blue) appear normally distributed around zero, the diagonals (yellow) are not. The diagonal and off-diagonal distributions differed significantly (t(102.8) = 25.09, p ≪ 0.001).

We hypothesised that these correlations would be higher when the observed and predicted maps came from the same subject, rather than from different subjects (Fig. 2a). We therefore took the inner product of all subjects' observed maps and all subjects' predicted maps, for the leave-one-out analysis, which produced a 103 × 103 matrix of correlations (Fig. 2b). In Fig. 2b, the rows and columns of this similarity matrix have been re-ordered to separate patients and controls, then row- and column-normalised. A strong diagonal visually indicates that the model predicts individuals well, and best matches predictions of individual subjects with their actual activation maps.

**Fig. 3:**
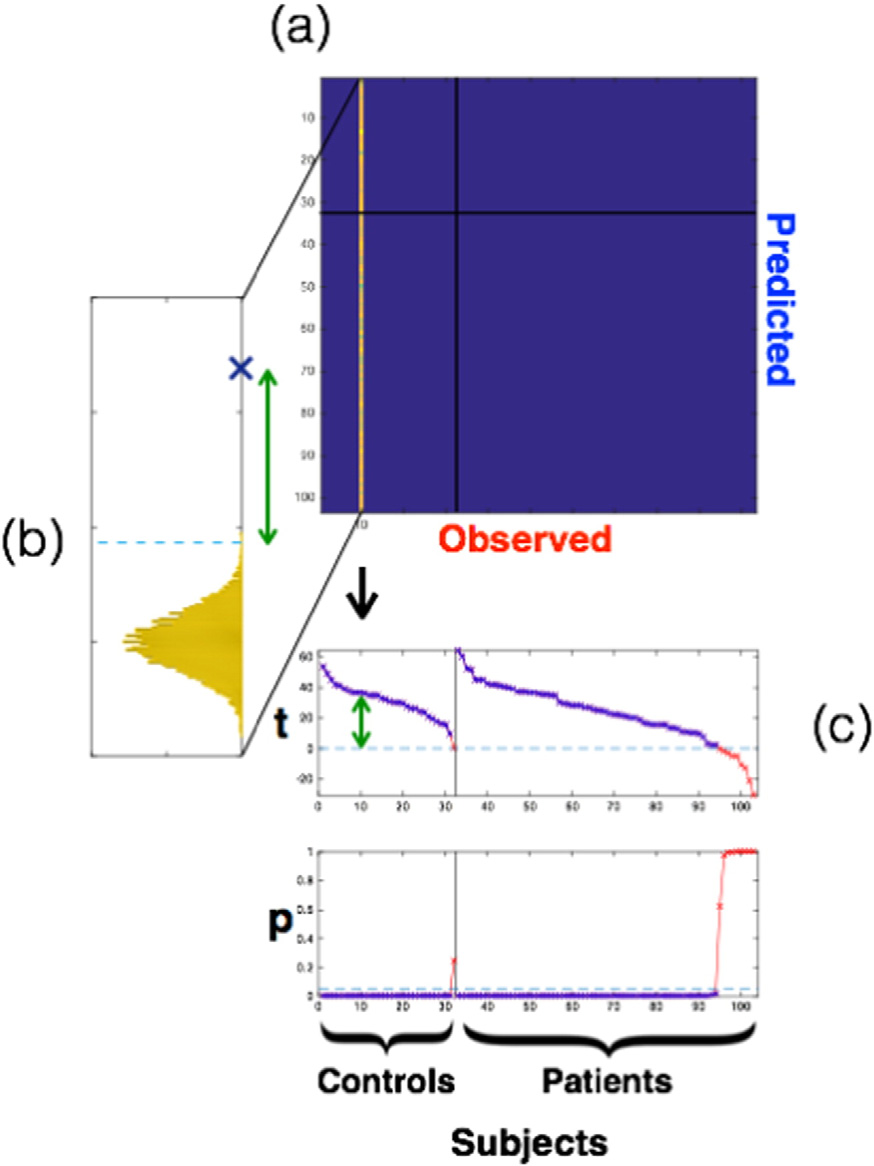
Significance Testing. To evaluate how well each subject's prediction (from resting fMRI) matched his or her observed activation map (from task fMRI), we constructed a statistical baseline. (a) The top panel shows the correlations for all subjects' predictions (rows) against one subject's observed task activation map, with all other subjects' data having been masked out in blue. (b) A baseline null distribution was constructed from all between-subject correlations (yellow histogram), where the diagonal element (blue ‘x’) represents the correlation between example subject's observed and predicted maps. Non-parametric *t*-tests were used to compare subject prediction against baseline using sign randomisation. We expected to find significant differences (green arrow) for models that distinguished between baseline and prediction. (c) The bottom panels report t-stats and p-values for all subjects with the results divided into controls (left) and patients (right). Significant values have been presented in blue and non-significant values in red. For the main analysis (leave-one-out), individual maps were better predicted than the baseline in 93/103 cases.

To quantify our ability to predict individual task activation maps, we computed a *t*-test for each subject's observed map. This compared the correlation with the same subject's prediction against the correlations with all other subjects' predictions (this is schematised below in Fig. 3). We built up a statistical test by first subtracting, for each subject, the diagonal from the extra-diagonal elements of the correlation matrix. We then used a non-parametric *t*-test with 10,000 sign-based permutations to generate a null distribution for each subject. This resulted in group-level t-statistics and p-values for each of the 103 subjects, where the significance level was set to p = 0.05.

In Fig. 2a we used thresholded binary images and calculated Dice coefficients of overlap (Dice, 1945).

### 2.8 Thresholding

Thresholding was used for visualising the results (see Fig. 1, Fig. 2, and Fig. 5) and did not affect any of the statistical results reported (e.g. correlations), which were performed on the full, unthresholded maps.

To visualise the results, we thresholded the task activation maps using a combination of cluster-masses and a mixture model.

The mixture model was a combination of one Gaussian and two Gamma distributions (Beckmann and Smith, 2004). The Gaussian was intended to fit the noise in each image so the Gammas could fit the positive and negative activations. As our interest was on the positive contrast task > baseline, we excluded values below the median of the upper Gamma distributions (see Supplementary Fig. 2).

Separate mixture models were fitted to each task and predicted map, producing 103 subject-specific thresholds for the task maps and 103 subject-specific thresholds for the prediction maps (this was also repeated for the leave-one-out and split analyses).

The mixture models were used to threshold images by activation height. To limit extent of activation in the visualisations, we used cluster mass thresholds (Bullmore et al., 1999, Hayasaka and Nichols, 2004). The mass of each cluster can be calculated as

$$c=\sum_{v\in K_{i}}T\left(v\right)$$

where **T**(*v*) is the (thresholded) statistical image (map), indexed by a set of vertices *v* in the cluster **K**_*i*_. Intuitively, the mass of a cluster is the sum of its vertex values. So a very narrow cluster with high values might have equivalent mass to a very wide cluster with low values. Clusters with masses below a set threshold were excluded from the visualisations. Signal strength was lower in the predicted maps. So, while a cluster-mass threshold of 240 was used for the observed task activation maps, we used a cluster-mass threshold of 120 for the predicted task activation maps. As this was constant across all subjects, there was no risk of biasing our visualisations of individual results by having different thresholds for predictions and observations.

### 2.9 Quality control

To evaluate data quality, we quantified in-scanner head movement and computed a temporal signal-to-noise ratio (tSNR) as a measure of changes to the MRI signal in time across the whole-brain (Murphy et al., 2007) for each scan (resting state and task data). We further used the movement parameters as estimated by MCFLIRT in combination with the task design to quantify stimulus-correlated motion. We correlated each of these three measures with the individual t-statistics described above to test whether model performance was related to the quality of the data (see Model evaluation).

### 2.10 Code availability

Code available upon request.

## 3 Results

In line with prior literature, the observed task activation maps (derived from task-evoked fMRI) showed pronounced activity in the left frontal lobe (Paulesu et al., 1997, Costafreda et al., 2006), with clusters that varied between subjects in shape, size, and strength. *Predicted* task activation maps (derived from resting-state fMRI) varied qualitatively between subjects in a similar way while closely resembling their intended target maps (Fig. 1).

**Fig. 4:**
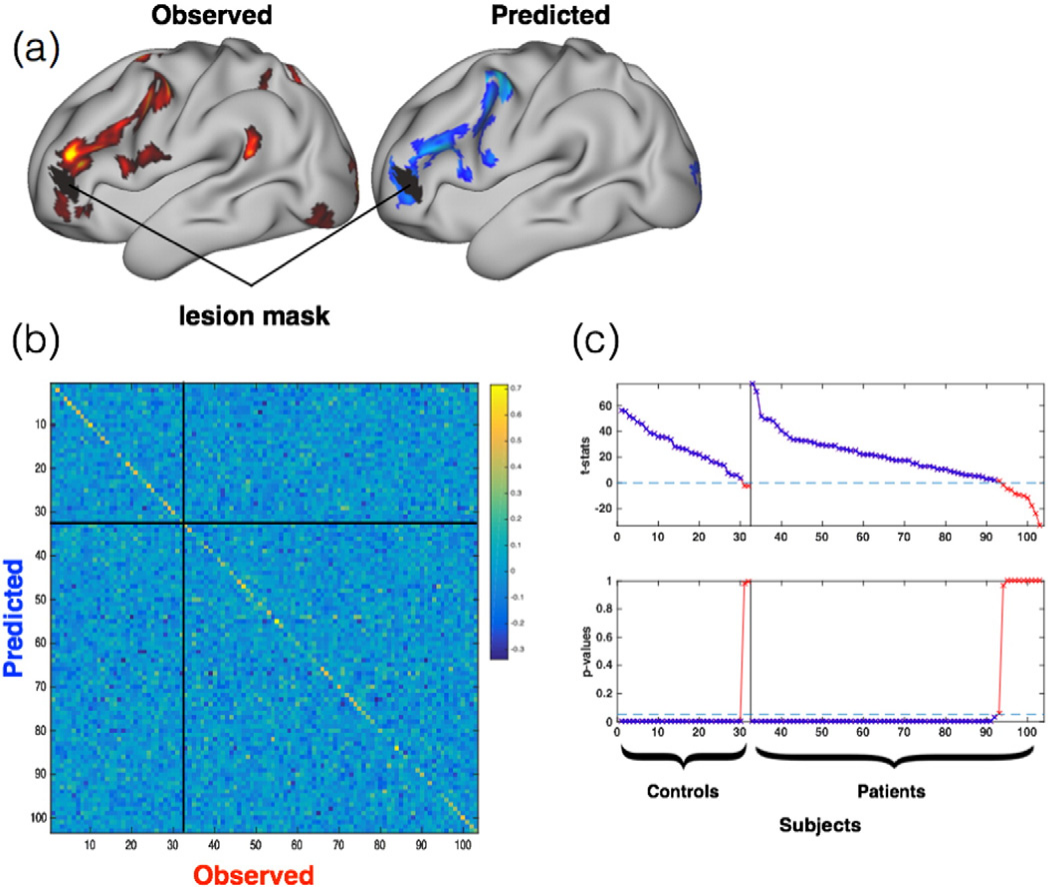
Correlations are not driven by lesions. a) A potential confound in the analyses of patient data is that the correlations between observed and predicted maps might be driven by lesions. This concern is motivated by the fact that lesions are the same in each subject's observed and predicted maps (shown here for one example subject with the lesion mask in black); and because little to no neural activity was observed within the lesion masks. To check that the results were not driven by lesions, we repeated the main analyses above while excluding the union of all lesion masks (see Supplementary Fig. 4). b) The lesions did not strongly affect the results, as shown by the similarity matrix (between observed and predicted language maps). For example, a heavy diagonal was again found in both controls (upper-left) and patients (lower-right quadrant). c) The repeated *t*-test analysis was very similar as before, with 90/103 individuals identified better than baseline (cf. 93/103). Of the three subjects previously identified but not identified here, two were control subjects. Consequently, removing the lesion masks resulted in a decrease in accuracy of only one patient.

We quantified these results by calculating correlations between all subjects' observed and all subjects' predicted maps (Fig. 2). We expected these correlations to be stronger within-subjects (‘matched’ predictions) than between-subjects (‘unmatched’) for any model capturing individual variability in task response. We therefore compared the matched and unmatched predictions for each subject, expressing the result as a t-statistic (Fig. 3; see Methods). The leave-one-out analysis showed that 90.3% of the matched predictions were significantly better than the unmatched predictions (i.e., p < 0.05 in 93/103 subjects). The analysis predicted task activity better in healthy controls (31/32, 96.9%) than in the more heterogeneous patient group (62/71, 87.3%), with the between-group difference being significant (t(101) = 2.13, p = 0.04). However, the type of pathology (epilepsy, tumour, or other) did not affect the accuracy of patient predictions (F(2,68) = 0.23, p = 0.79). A potential confound was that subject-specific lesions were common to both observed and predicted maps, and might therefore have driven the subject-wise correlations. To address this, we re-ran the analyses above after excluding the union of all patient lesions (a large binary mask) from all subjects' data. We found that lesions did not explain the predictive ability reported above Fig. 4.

To understand why some individuals were better predicted than others, we ran post-hoc correlations on individual t-statistics and three quality-control measurements: (i) task-correlated in-scanner motion, (ii) task tSNR, and (iii) rest tSNR. The correlations reveal individual predictions degrading with the quality of the resting-state scans (r = 0.23, p = 0.02). Behavioural or demographic measures, such as sex, age, or out-of-scanner behaviour, did not significantly explain model performance (Supplementary Table 1).

**Fig. 5:**
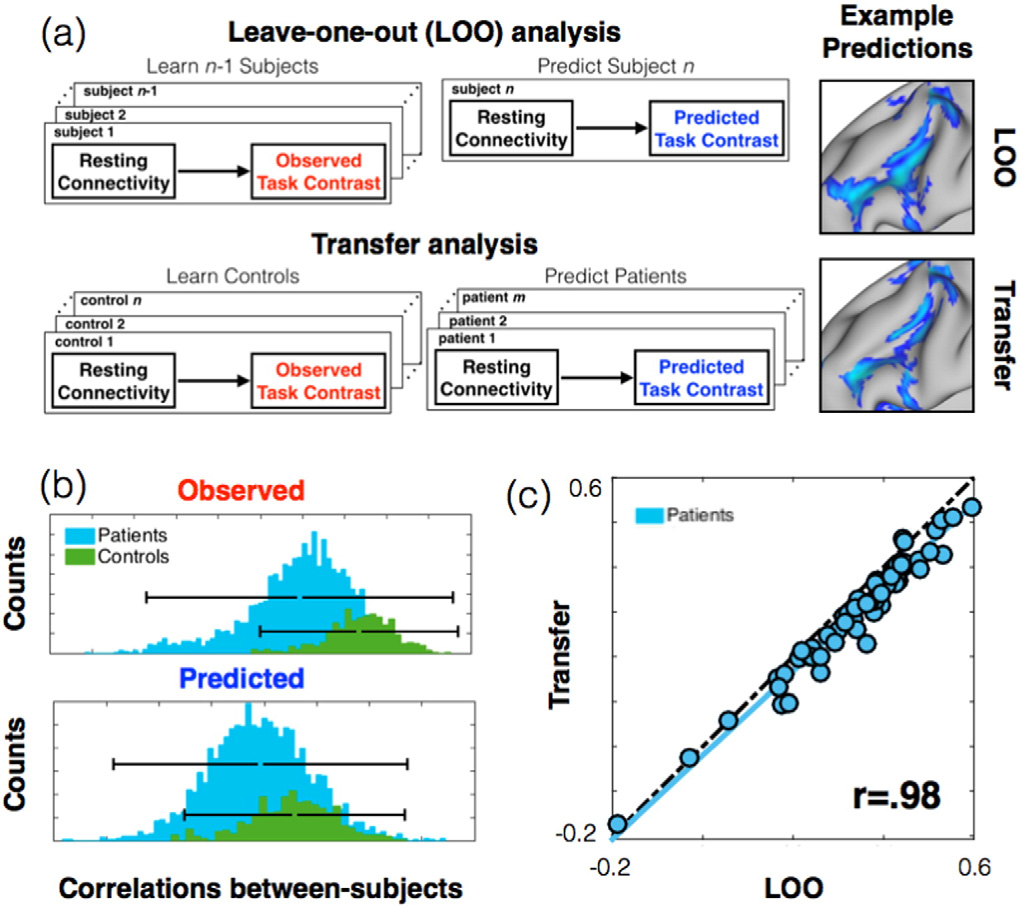
Training on controls, predicting patients. (a) Schematics for the two analyses. Above: in the leave-one-out (LOO) analysis, data for one subject was removed from the training set and used to evaluate the model prediction. The procedure was then repeated for each subject, resulting in 103 subject models and predictions. Below: we performed a one-time split of data into patient and controls groups in the transfer analysis. Using the control data, we trained a single model that was used to make predictions for all patients. Example predictions are shown (to the right of each schematic) for one subject (patient (d) from Fig. 1). The examples show that the two analyses produce qualitatively similar predictions. (b) Histograms of correlations between subject maps show greater variability in the patient group. Both in the observed task-activation maps (upper panel, red) and in the predicted task-activation maps (lower panel, blue), patients varied more than controls. The solid bars show three standard deviations from the distribution means. The results here are from the LOO analysis, because the transfer analysis applied only to patients. (c) Scatter plot showing the correlation in patients between observed and predicted task maps (dotted line shows x = y; light blue line shows the best liner fit). The predicted maps were nearly identical in LOO and transfer analyses (r = 0.98). The transfer analysis therefore predicted variability in patients virtually as well as the leave-one-out analysis did, even though the transfer-analysis was not trained on a single patient.

The results of the leave-one-out and transfer-learning analyses were nearly identical for patients. This is important because it means that a model trained on a relatively homogenous group (controls) can extrapolate its predictive power to predict the neural responses observed in a more variable group (patients) (see Fig. 5). Indeed, the larger variability in patients compared to controls was seen in both observed activation maps but also in our model predictions (Fig. 5b), which illustrates the power of our model to extrapolate from controls to patients. In practical terms, this result means that additional models might be trained on one group (healthy volunteers) and then applied to another group (patients) without the need for the latter group to perform any additional experiments. Repeating the evaluation procedure above, we found that the transfer-learning analysis predicted 84.5% of the patients better than baseline (60/71). This was only two patients fewer than the leave-one-out analysis (62/71) and the similarities between predicted and actual maps from the two analyses were very highly correlated (r = 0.98, see Fig. 5). Because each subject's predicted task maps resulted from only two pieces of information—(i) the model parameters, and (ii) model input (connectivity features)—and because the model parameters were held constant between all patients in the transfer-learning analysis, we conclude that the variability observed between-subjects was driven by differences in the individual connectivity features.

## 4 Discussion

Using clinical-grade neuroimaging data and a regression-based machine learning approach, we found that between-subject variations in observed language maps, inferred from task-based fMRI, were closely predicted by individual measures of resting connectivity both in healthy controls and in pre-surgical patients (Fig. 1, Fig. 2). The predictions were marginally better in controls, which is unsurprising given that greater variability is observed in patients (Fig. 5b). Importantly, the model correctly predicted there to be higher variability in patients (as seen in the actual activation maps). Moreover, we were able to predict the more highly variable patient maps from a model trained only on homogenous controls. These *transfer-learning* predictions were almost identical to the ones obtained in models trained on both patients and controls (Fig. 5c).

Many studies have previously pointed to a relationship between task activation maps and resting connectivity (e.g. Smith et al., 2009, Cole et al., 2014), suggesting the existence of an ‘intrinsic’ architecture of functional organisation. This relationship was previously exploited to understand and model individual variations in task-activations across a wide range of behavioural domains, including a receptive (as opposed to expressive) language task (Tavor et al., 2016). It has relatedly been shown that resting-state connectivity can be used to identify individuals (Finn et al., 2015) and predict their behavioural performances (Rosenberg et al., 2016). Yet the present paper is the first to explore the relationship of resting-state connectivity and task fMRI beyond young and healthy control subjects, specifically in the more highly-variable cases of patients diagnosed with drug-resistant epilepsy, brain tumours, and vascular abnormalities.

The emphasis on these patient populations was motivated by the potential use of resting-state fMRI for pre-surgical planning. Because damage to areas in the brain that support key functions like language may cause lasting aphasias and other deficits, the identification of these regions plays a central role in surgical planning (Ojemann et al., 1989). As noted in the introduction, language mapping with fMRI is often limited by patient fatigue, linguistic ability, or patient compliance, and clinicians are typically constrained by time to probe a limited number of task contrasts. Consequently, the ability to identify critical brain regions potentially involved in multiple behavioural domains (Tavor et al., 2016) from short resting scans, which do not place active demands on patients and which are readily available on all modern MRI scanners, would provide a valuable alternative strategy when no other options are available.

Although we found our results to be highly robust across subjects (Supplementary Fig. 3), they were not perfect. When we correlated prediction performance with behaviour, demographic information, and quality control measures from the scanner, we discovered that at least part of the predictive shortcomings could be attributed to poor-quality resting scans (Supplementary Table 1). This result is useful because scan-quality can be assessed before using these models. In future work, we plan to develop additional quality control measures on the fMRI data, to assess the applicability of a trained model and suitability of predictions.

Although we know that the quality of the model predictions can be further improved by increasing scan duration and temporal sampling times (Smith et al., 2013, Tavor et al., 2016), it was nonetheless remarkable to achieve the present results using only 5 min of resting data. This speaks to the robustness and potential clinical use of the method. We deliberately did not attempt to optimise the feature extraction step or the parcellation that feeds into the piecewise linear model and instead employed the same approach used by Tavor et al. (2016). The main reason for this is that we did not wish to overfit the model to this particular cohort of subjects. Future work might nonetheless improve upon the results by using nested cross-validation to optimise the feature selection and parcels on a separate validation set.

Further improvements to the resolution and accuracy of the predicted maps might be gained by incorporating multi-modal data from other sources, such as diffusion tractography (Saygin et al., 2012, Osher et al., 2016). However, before any predictive method is used in a clinical setting, it will be imperative to perform more validation studies.

Here we used task-evoked fMRI as ‘ground truth’ of neural activation patterns. Alternative behavioural ‘ground truths’ include direct cortical stimulation and post-operative neurocognitive outcomes (Penfield and Roberts, 1959, Ojemann et al., 1989). Ultimately, for clinical applicability, it will be important to determine how well model predictions foretell intra- or post-operative behavioural outcomes in comparison to task fMRI. Despite their use in pre-surgical planning, fMRI-based language maps are liable to produce false positives (apparent activation in regions where surgical damage would not result in significantly different cognitive or behavioural outcomes) and false negatives (apparent lack of activity where surgical intervention could result in aphasias). Therefore a combination of direct cortical stimulation and subject testing is advocated wherever possible as a final check before excising brain tissue during surgery.

To summarise, more work will be needed to validate and build on our method before it may be used in clinic, but we hope that the present results, showing that predictions based on resting fMRI can reasonably approximate task-evoked language maps in both patients and controls, will have laid a solid foundation.

## Author contributions

N.L.V., J.E.A., and R.S. acquired the data; O.P.J. and S.J. performed the analyses; O.P.J., N.L.V., and S.J. wrote the paper.

## Competing financial interests

The authors declare that they have no competing financial interests.

## Figures and Tables

**Table 1:** Subject details. Thirty-two of 103 subjects were healthy controls (CON). The remaining 71 represented five neurosurgical conditions: temporal lobe epilepsy (TLE); brain tumour (TUM); cavernoma (CAV); arteriovenus malformation (AVM); and focal cortical dysplasia (FCD). Wada test results are expressed as a real number ranging from − 1 (right-hemisphere dominant) to 1 (left-hemisphere dominant). Fluency results reflect the total number of vegetables the subjects named overtly, out-of-scanner in 1 min.

	Subject group					
	CON	TLE	TUM	CAV	AVM	FCD
n	32	42	20	5	3	1
Sex	17 male	20 male	10 male	1 male	2 male	0 male
Age:						
Range	19–48	16–60	14–65	30–59	20–32	17–17
Mean	32	35	40	44	28	17
Handedness:						
right	27	37	19	4	1	1
left	4	5	1	1	0	0
both	1	0	0	0	2	0
Wada:						
n	–	29	3	1	–	–
Mean	–	0.6	0.7	1	–	–
Fluency:						
n	–	41	10	–	–	–
Mean	–	20	20	–	–	–
R0061nge	–	8–44	9–28	–	–	–

## Appendix A Supplementary material

Supplementary material Image 1
