# Supplementary material for: Resting connectivity predicts task activation in pre-surgical populations
Source: Neuroimage Clin. 2016 Dec 24;13:378–85. doi: 10.1016/j.nicl.2016.12.028 (PMC5222953; doi:10.1016/j.nicl.2016.12.028)
Supplement: Supplementary file 1 — Supplementary material [file mmc1.docx]

# Supplemental Information


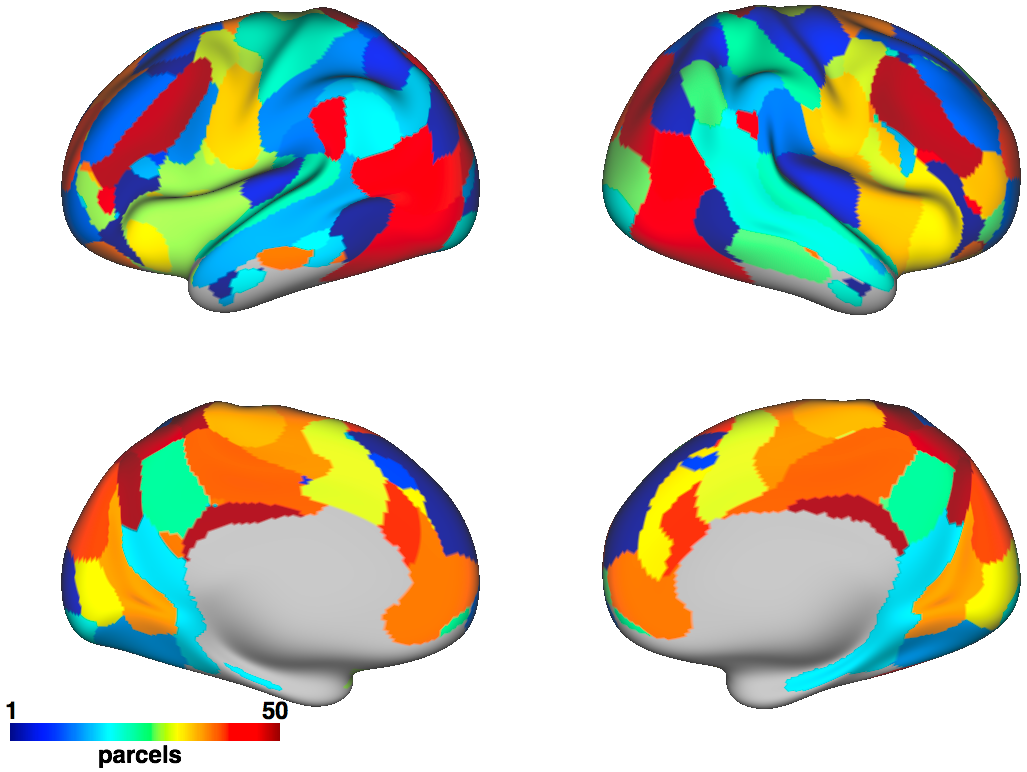


Supplementary Figure 1: Parcels. Rather than directly fit one model per brain, we found it useful to divide the brain surface into parcels, and then fit a model on each parcel. The results could then be concatenated to produce the whole-brain prediction without loss of resolution. The parcels were found using group ICA with 50 components on an independent sample of 100 subjects from the HCP. We applied a ‘winner-takes-all’ heuristic to each vertex so that it was associated with only one component, which was the one it most strongly represented.


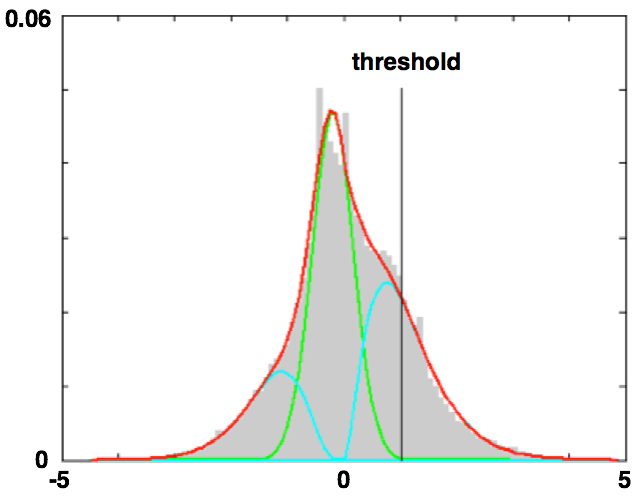


Supplementary Figure 2: Thresholding. We used a mixture model to threshold the raw task activation maps. The model fitted a Gaussian (green) and two Gamma (cyan) distributions to a histogram of whole-brain activation strengths. In this study we focused on the positive Gamma distribution (positive activation). The median of this distribution was automatically selected for each subject (see vertical bar labelled ‘threshold’). For illustrative purposes, we removed activity below this median threshold in the maps. In addition we applied a second threshold (cluster-mass) using a mass of 240 for all observed and 120 for all predicted maps.


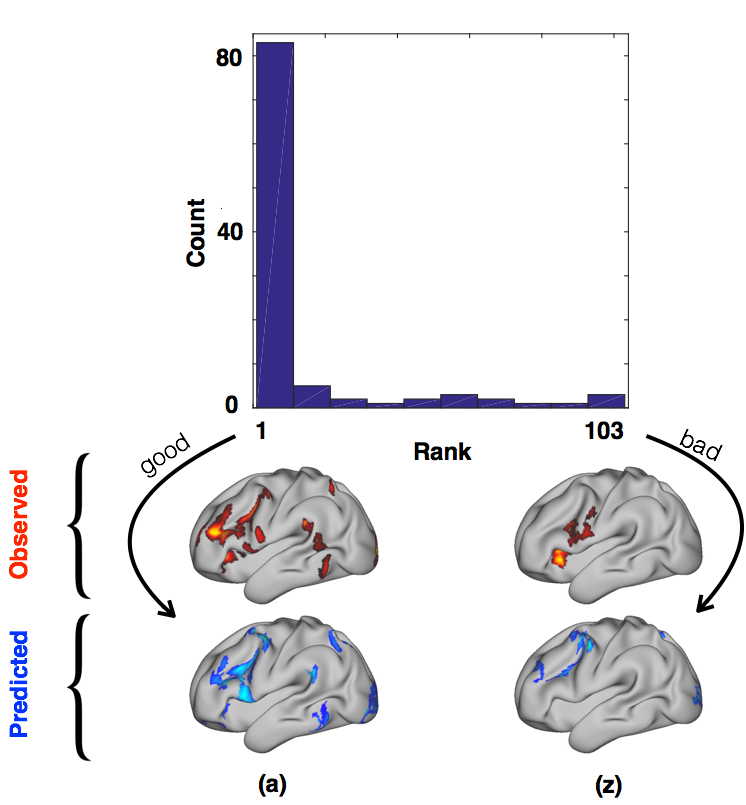


Supplementary Figure 3: Reliability of results. For illustrative purposes, we naturally picked strong results in the examples above (see e.g. Figure 1). However this leaves open the question of how reliable the results were in general. Were the illustrative example representative? Here we summarise the results for all subjects, depicting both the best (a) and worse (z) predictions along with the distribution of every other result in between. Goodness was established by sorting the raw similarity measures between observed and predicted maps (see (a) in Figure 3). We defined rank as the position of the individual prediction (diagonal element) within the list of sorted predictions. For example, if the individual’s prediction was *better* than all other predictions, it received a rank of 1; if it was *worse* than all others, it received a rank of 103 (out of 103). The histogram of ranks is strongly weighted toward 1. This shows that while we did occasionally generate a weak prediction (see subject (z)) the vast majority of predictions were reliably good. Concretely, 83 subjects were in the top 10 (bin size above)—and of these, 64 had ranks of 1, seven had ranks of 2, and one had a rank of 3.


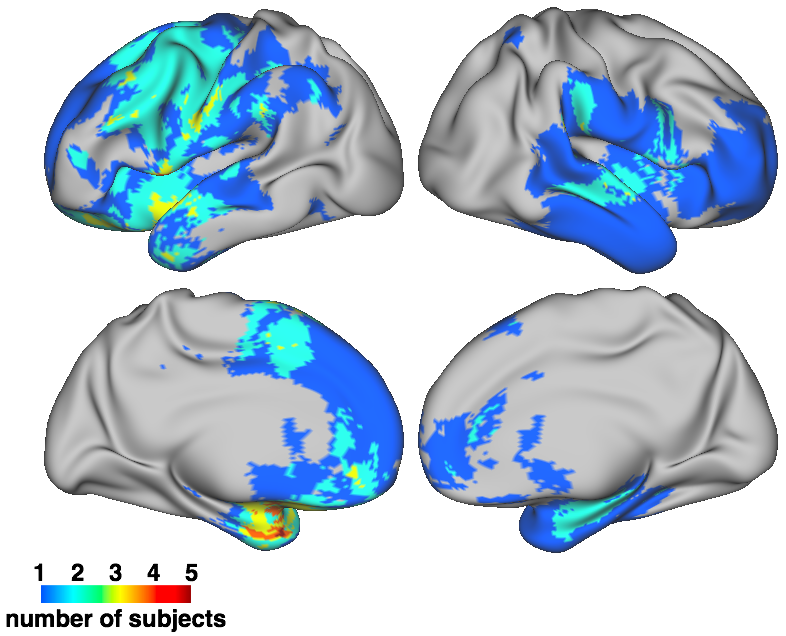


Supplementary Figure 4: Lesion overlap maps. 28 patients had lesion masks caused by AVM, cavernoma, or tumour. Lesions were manually defined on the T1w anatomical scan and projected onto the brain surface. Lesions covered large extents of frontal, temporal, and parietal lobes. Maximum overlap (for 5/28 subjects) was found in the left temporal lobe.


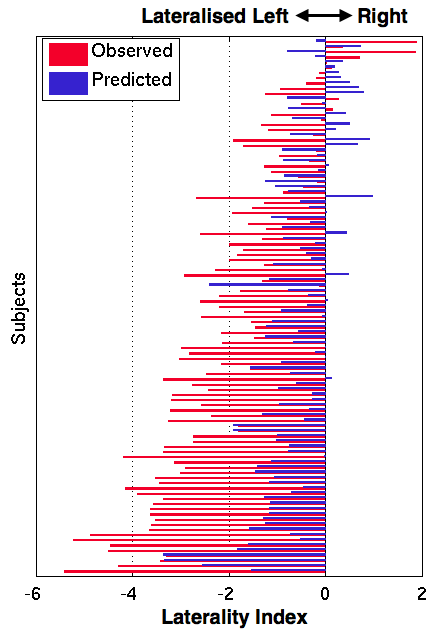


Supplementary Figure 5: A laterality index (difference between right and left activation levels) was calculated for each subject’s observed and predicted maps (red and blue, respectively). In general, the model was able to predict lateralisation (r(101)=0.28, p=0.004) even though most subjects were left-hemisphere dominant or bilateral for language (all potential right-dominant exceptions were patients). In a recent study, with more data per subject, we could predict laterality indices that were more faithful to observed laterality indices (see Tavor et al. 2016).


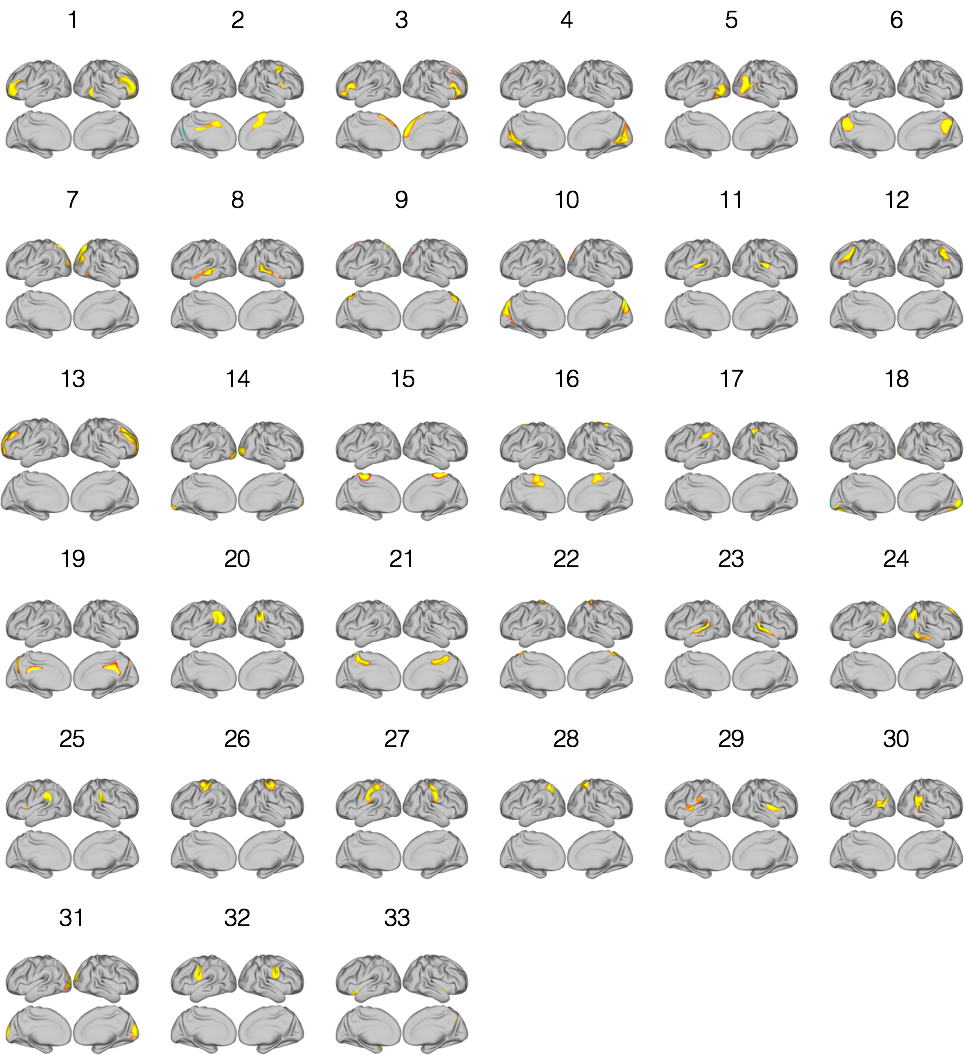


Supplementary Figure 6: Group ICA components. Resting connectivity features were obtained from a separate cohort of 100 subjects drawn from the HCP. These group-level ICA components were used to derive individual resting-connectivity features using dual regression in the clinical dataset, and the individual connectivity features were then used in the piecewise linear model to predict the same individuals’ task contrasts, language maps for the covert fluency task.

|  | r | p | n |
| --- | --- | --- | --- |
| sex | ~0 | .07 | 103 |
| age | 0.05 | 0.65 | 103 |
| hand | -0.1 | 0.3 | 103 |
| Wada | -0.04 | 0.83 | 70 |
| CAT | 0.13 | 0.37 | 51 |
| movement | -0.06 | 0.53 | 103 |
| task tSNR | 0.04 | 0.71 | 103 |
| rest tSNR | 0.23 | 0.02* | 103 |

Supplementary Table 1: Correlations for all demographic, behavioural, and quality control measures with individual prediction (t-stat). Asterisk: only the correlation with rest tSNR was significant.
